# Gendered Development of Motivational Belief Patterns in Mathematics Across a School Year and Career Plans in Math-Related Fields

**DOI:** 10.3389/fpsyg.2019.01472

**Published:** 2019-06-28

**Authors:** Julia Dietrich, Rebecca Lazarides

**Affiliations:** ^1^Institute of Educational Science, University of Jena, Jena, Germany; ^2^Department of Education, University of Potsdam, Potsdam, Germany

**Keywords:** motivation in mathematics, latent transition analysis/latent profile analysis, expectancy-value theory, heterogeneity, adolescence

## Abstract

Rooted in Eccles and colleagues’ expectancy-value theory, this study aimed to examine how expectancies and different facets of task value combine to diverse profiles of motivational beliefs, how such complex profiles develop across a school year, and how they relate to gender and career plans. Despite abundant research on the association between gender and motivational beliefs, there is a paucity of knowledge regarding the gendered development of student motivational belief profiles in specific domains. Using latent-transition analysis in a sample of *N* = 751 ninth to tenth graders (55.9% girls), we investigated girls’ and boys’ development of motivational belief profiles (profile paths) in mathematics across a school year. We further analyzed the association between these profile paths and math-related career plans. The results revealed four motivational belief profiles: high motivation (intrinsic and attainment oriented), balanced above average motivation, average motivation (attainment and cost oriented), and low motivation (cost oriented). Girls were less likely than expected by chance to remain in the high motivation profile, while the opposite was true for boys. The math-relatedness of students’ career plans was significantly higher in the “stable high motivation” profile path than in all other stable profile paths.

## Introduction

Motivation declines during adolescence, especially in STEM (science, technology, engineering, and math) subjects. This decline regards, for instance, math interest/intrinsic value (Frenzel et al., 2010; Dietrich et al., 2015), the perceived usefulness of math (Watt, 2004), and perceived competence in math (Watt, 2004). Although girls’ and boys’ motivation similarly declines across adolescence (Jacobs et al., 2002), girls report lower levels of mathematics interest (Frenzel et al., 2010) and competence beliefs (Watt, 2004) than boys. Such gendered motivational beliefs in math and other STEM fields are related to gendered career plans (Lauermann et al., 2017; Lazarides et al., 2017).

Most existing research that focused on the development of girls’ and boys’ motivational beliefs and the relations of such beliefs with career plans has been variable-centered. One limitation of this approach is the underlying assumption that the associations between gender, motivational beliefs and career plans are similar across the whole continuum of low to high motivation. Furthermore, it remains unknown how expectancies and the different value facets combine to different patterns of motivational beliefs, how such complex patterns develop over time, and how they relate to gender and career plans. The present study aimed to overcome some of these limitations.

We focus on adolescents’ motivational beliefs about mathematics as defined in Eccles and colleagues’ expectancy-value theory (EVT) of achievement motivation (Wigfield and Eccles, 2000). The EVT seeks to explain achievement-related (e.g., career) choices through expectancies for success and subjective task values. In this study, we operationalized the expectancy component as a student’s academic self-concept, defined as the subjective beliefs about one’s abilities in mathematics (Marsh and Martin, 2011). In line with EVT, the value component is differentiated into interest (intrinsic value), usefulness for future goals (utility value), personal importance (attainment value) and perceived costs of learning mathematics (cost value) (Wigfield and Eccles, 2000). Expectancies and values are conceptualized as correlated but independently functioning in predicting achievement-related behaviors. Moreover, the theory predicts variation in the relative salience of different value facets (Eccles, 2005).

Indeed, research suggests that the relations between different motivational beliefs are heterogeneous in the student population. Corresponding with findings from the correlational literature, many classification-based studies report patterns of overall high, moderate, and low motivation (Viljaranta et al., 2017; Lazarides et al., 2019). Going further, patterns of mixed motivation have been found, such as high self-concept combined with low interest (Lazarides et al., 2019), and high interest combined with high perceived cost of doing well in math (Conley, 2012).

Only few studies have addressed changes in motivational profiles using longitudinal data (in the following labeled “profile paths,” e.g., Marcoulides et al., 2008; Lazarides et al., 2019). These studies suggest that patterns of motivation are relatively stable in adolescence with probabilities around 0.70–0.90 of staying in the same profile. But that also means that some adolescents do exhibit profile changes, even during a very short time span (e.g., Martinent and Decret, 2015). Building on these previous studies we adopted a longitudinal classification-based research strategy. This allows to discern the extent to which the development of students with low motivation profiles differs from the development of students with high motivation profiles and from that of students with mixed motivation profiles. Our first two research questions were:

(1) Which kinds of expectancy-value patterns or profiles of math motivation can be identified at two time points in the school year? We expected to find high, moderate, low, and mixed motivation profiles.
(2) How many students change their motivation profile across the school year? We hypothesized profile stability to be typical with only some students changing their profile of math motivation.

Abundant research has shown that gendered pathways into and away from STEM fields are mediated through motivational beliefs (Eccles and Wang, 2015; Lauermann et al., 2017). Even in the case of equal achievements in mathematics, girls find mathematics less interesting (Frenzel et al., 2010), perceive lower job utility of mathematics (Gaspard et al., 2015), and feel less competent compared to similarly achieving boys (Marsh and Yeung, 1998). The few studies investigating gendered patterns of motivation in STEM fields showed that boys dominated the high-math-and-science profiles and the group with high mathematics self-concept, but low interest, while girls dominated the low-math-and-science profiles (Chow and Salmela-Aro, 2011; Lazarides et al., 2019). Studies are largely missing that would investigate *gendered changes in patterns* (i.e., longitudinal profile paths) of motivational beliefs and relations between such profile paths and plans for math-intensive careers. Such studies would add more detail to the associations of motivation with gender and with career plans. In adolescence, girls and boys face developmental changes such as a greater importance of gender identity development and closer peer relations (Erikson, 1959). Interests in school subjects are a means to communicate self-identity (Kessels, 2005), which might be particularly relevant to motivation in school subjects which are typically stereotyped as “male,” such as math and physics (Nosek et al., 2002). Research has shown that physics-oriented girls are less popular than are their peers, because they behave in opposition to the female stereotype (Kessels, 2005). Also in math, girls might feel pressured to behave in gender-typical ways and face a greater likelihood than boys of changing their motivational profile, especially when they are highly motivated.

Our third and fourth research questions were:

(3) In which longitudinal profile paths (stable profile paths and profile changes) are gender disparities especially prevalent? We expected girls (boys) to be under(over)represented in stable high motivation paths and over(under)represented in stable low motivation paths. We moreover expected a greater likelihood for girls to change their motivation profile.
(4) Which longitudinal motivation paths are most strongly related to plans for math-intensive careers? We expected that the career plans of students with stable high motivation would evidence the highest mathematics-relatedness, compared to other motivation paths.

## Method

Data stem from two waves of the German longitudinal Motivation for Learning Mathematics study (Lazarides and Rubach, (2015-2017)). The Berlin Senate for Education, Youth, and Research approved the study. An ethics approval was not required at the time the study was conducted as per the then applicable institutional and national guidelines and regulations. The participating students and their parents gave written informed consent. For the present analyses, data from 751 ninth to tenth graders (55.9% girls; 71.2% native speaker) in two school types were used (academic track schools, “Gymnasium”: 53.8%; integrated secondary school, “Integrierte Sekundarschule”: 46.2%). Data were assessed 2 months after the beginning of the school year (Time 1) and again 6 months later (Time 2). Supplementary Appendix A shows descriptive statistics for all variables.

*Mathematics task values* were assessed with four subscales: Intrinsic value (e.g., “I like doing math”), utility value (e.g., “Math content will help me in my life”), and attainment value (e.g., “It is important to me to be good at math”) were assessed with three items each (adapted from Steinmayr and Spinath (2010), 1 = *does not apply at all* - 5 = *fully applies*). Cost value was also assessed with a three-item scale comprising effort cost, emotional cost, and opportunity (e.g., “Doing math is exhausting to me”, effort cost) based on Gaspard et al. (2015). Cronbach’s alpha reliabilities at T1/T2 were 0.92/0.92 for intrinsic value, 0.90/0.92 for attainment value, 0.87/0.89 for utility value, and 0.79/0.78 for cost value.

*Self-concept in mathematics* was assessed with four items (e.g., “I think I am … in mathematics” from “1 = *not talented* - 5 = *very talented;* (Steinmayr and Spinath, 2010)). Reliabilities at T1/T2 were 0.87/0.88.

*Mathematics-related career plans* were assessed with the item “What job would you like to have in the future?” Students’ open-ended answers were coded for the mathematics-relatedness of the nominated career using the Occupational Information Network (O^*^NET; National Center for O^*^NET Development, 2014) to quantify relatedness to “knowledge of arithmetic, algebra, geometry, calculus, statistics, and their applications” on a scale ranging from 0 = *not mathematics-related* to 100 = *completely mathematics-related*.

## Results

We used Latent Transition Analysis (LTA; Nylund-Gibson et al., 2014) to examine research questions 1 and 2. Means and variances of the motivation variables (profile indicators) were allowed to vary across latent classes. We imposed measurement invariance with equal means and variances in a given latent class over time. We statistically evaluated the appropriate number of latent classes based on information criteria (BIC, aBIC, AIC). Theoretical interpretation and the number of cases per class were also used for model selection (Berlin et al., 2014).

**FIGURE 1:**
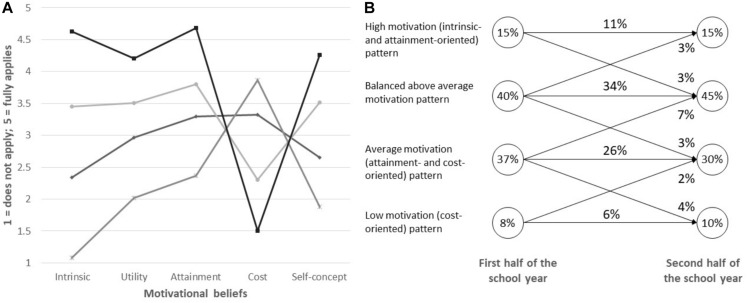
**(A)** Means across all motivational beliefs for the four-profile solution. **(B)** Profile sizes (proportions for the latent class variables: circles) and profile changes (proportions for the latent class patterns: arrows) based on the estimated LTA model. Circles depict the percentage of the sample in a given profile in the first/the second half of the school year. Arrows depict the percentage of the sample that shifts between the two profiles linked by each arrow.

We identified the four-class model (Figure 1) as the best fitting LTA model (see Supplementary Appendix B). Pattern 1 was characterized by particularly high intrinsic and attainment value and low perceived cost and was labeled as “*high motivation* (*intrinsic and attainment oriented*)” pattern. Pattern 2, labeled “*balanced above average*” motivation, was similar to pattern 1, but the levels of motivational beliefs were one scale point closer to the mid of the scale. Pattern 3 was characterized by motivation levels around the scale midpoint, with highest levels on attainment and cost values, and was labeled “*average motivation* (*attainment and cost oriented*).” Pattern 4 was characterized by low intrinsic, attainment and utility values, low self-concept, and high costs and was labeled “*low motivation* (*cost oriented*)” pattern. As hypothesized, profiles were highly stable with 77% of students remaining in their motivational pattern across the school year. The most frequent change was from “average” to “balanced above average” motivation (7% of students). Other changes are depicted in Figure 1, and the transition probabilities are depicted in Supplementary Appendix C.

For further analyses involving gender and career plans (research questions 3 and 4) we saved each individual’s most likely latent class as a manifest variable (the classification probabilities ranged 0.70 to 0.97, *M* = 0.81). In doing so, we used the latent class patterns which refer to each individual’s longitudinal profile path (e.g., stable high motivation or moving from “average” to “low” motivation).

We used Configural Frequency Analysis (Heine and Alexandrowicz, 2015) to examine the association between gender and the longitudinal profile paths. Based on a cross-tabulation of profile path by gender, ConFA provides a test for each cell indicating whether this cell contains more or fewer individuals than expected by chance. We used the *z*-Test with Bonferroni correction (alpha = 0.002) for the cell-specific significance tests. There was a significant overall association between gender and profile path (Table 1). Cell-specific tests indicated that girls were less likely than expected by chance to remain in the ‘high motivation profile’ across the school year. The opposite was true for boys. Additionally, we tested whether girls were more likely to change their profile (change: 97 girls, 62 boys; stability: 323 girls, 269 boys), but that was not the case, χ^2^ (1) = 2.11, *p* = 0.146.

**TABLE 1:** Cell-Wise associations between longitudinal profile paths and student gender (ConFa).

**Profile path**		**Observed**	**Expected**	**z**	***P***
**Girls**					
High	High	28.00	48.35	–2.93	0.002
	Balanced	12.00	14.62	–0.68	0.247
	Average	0.00	0.00	0.00	0.500
	Low	0.00	0.00	0.00	0.500
Balanced	High	8.00	11.24	–0.97	0.167
	Balanced	138.00	150.12	–0.99	0.161
	Average	9.00	7.31	0.63	0.266
	Low	0.00	1.12	–1.06	0.144
Average	High	3.00	1.69	1.01	0.156
	Balanced	36.00	28.11	1.49	0.068
	Average	133.00	109.64	2.23	0.013
	Low	20.00	17.43	0.62	0.269
Low	High	0.00	0.00	0.00	0.500
	Balanced	1.00	1.12	–0.12	0.453
	Average	8.00	4.50	1.65	0.049
	Low	24.00	24.74	–0.15	0.441
**Boys**					
High	High	58.00	37.65	3.32	0.000
	Balanced	14.00	11.38	0.78	0.219
	Average	0.00	0.00	0.00	0.500
	Low	0.00	0.00	0.00	0.500
Balanced	High	12.00	8.76	1.10	0.136
	Balanced	129.00	116.88	1.12	0.131
	Average	4.00	5.69	–0.71	0.239
	Low	2.00	0.88	1.20	0.115
Average	High	0.00	1.31	–1.15	0.126
	Balanced	14.00	21.89	–1.69	0.046
	Average	62.00	85.36	–2.53	0.006
	Low	11.00	13.57	–0.70	0.243
Low	High	0.00	0.00	0.00	0.500
	Balanced	1.00	0.88	0.13	0.447
	Average	0.00	3.50	–1.87	0.031
	Low	20.00	19.26	0.17	0.433

**Note*. Overall test of association between gender and profile path: χ^2^ (15) = 54.41, *p* < 0.001. Bonferroni corrected alpha for the cell-specific *z*-tests = 0.002.*

In examining the relation between profile paths and career plans we focused on the stable paths (high – high, *n* = 86; balanced – balanced, *n* = 267; average – average, *n* = 195; low – low, *n* = 44) due to the relatively large sample sizes in these profile paths compared to profile changes (<1 to 7% of students). We conducted ANOVAs to test for differences in math-related career plans between the four profile path groups, and found significant overall effects both in the first half [*F*(3,400) = 11.19, *p* < 0.001] and in the second half of the school year [*F*(3,389) = 11.23, *p* < 0.001]. *Post hoc* comparisons (Tukey HSD test, Supplementary Appendix D) indicated that the math-relatedness of students’ career plans was significantly higher in the “stable high motivation” path than in all other profile paths. The math-relatedness of the career plans in students with “stable low motivation” did not differ from the math-relatedness in students with “stable balanced” or “stable average” motivation.

## Discussion

This study aimed to look beyond main-effects variable-(correlation)-centered models to study the interrelations of gender, career plans, and change and stability in profiles of motivational beliefs. We found profiles reflecting mainly level-differences which correspond to those shown in previous studies (Viljaranta et al., 2017; Lazarides et al., 2019). While these results converge with a variable-centered perspective on motivational beliefs, they additionally show that, for example, students with low motivation are especially low on intrinsic value, compared to their utility and attainment values (see also Eccles, 2005). Contrary to our expectations and to some previous studies (e.g., Conley, 2012) we did not find “mixed motivation” profiles. It might be that such profiles are more prevalent among primary school (Viljaranta et al., 2017) and younger secondary school students (Conley, 2012) than among ninth and tenth graders (Lazarides et al., 2019).

Going beyond previous studies, we were able to show some gender disparities in profile development. Boys were more likely to remain in the “high motivation (intrinsic and attainment oriented)” profile across the school year, while such a stable high motivation path was untypical for girls. These results are relevant as this stable high motivation path was in turn associated with the highest levels of math career plans, with differences in career plans getting even larger across the school year (Hedges’ *g* increasing by 0.12/0.15 for the differences between stable high and stable average/stable balanced motivation). Interestingly, gender differences were not evident in the stable low profile path.

Some of our results are in contrast to a previous study of Lazarides et al. (2019) who found no gender differences for stable high motivation from Year 9 to Year 10, but did find differences for stable low motivation such that girls were more likely to remain in that profile compared to boys. Other studies on the development of motivational profiles in adolescents (i.e., Alexander and Murphy, 1998; Nurmi and Aunola, 2005; Viljaranta et al., 2016) did not consider the role of student gender for such developmental changes. There is thus a need for more studies on these developmental aspects from a holistic, person-oriented perspective, and the special value of the present study is to help understand how gender differences in motivation evolve in different groups of students.

Important limitations of this study pertain to its reliance on self-report data (e.g., no achievement data were assessed) and the short time span studied. It might be that because stability was so high in this study, we did not find more frequent profile changes among girls as expected.

Overall, a research focus on the development of motivational profiles is worthwhile to capture the heterogeneity within and between students: it is very unlikely that every person develops in the same way (Molenaar, 2004). Accordingly, the results of this study suggest that the associations between (the development of) motivational beliefs, gender, and career plans vary across different levels and patterns of motivation.

## Ethics Statement

The Berlin Senate for Education, Youth, and Research approved the study. An ethics approval was not required at the time the study was conducted as per the then applicable institutional and national guidelines and regulations. All subjects gave written informed consent in accordance with the Declaration of Helsinki.

## Author Contributions

Both authors analyzed the data from a research project (MOVE) designed and conducted by RL and jointly contributed to the writing of the article.

## Conflict of Interest Statement

The authors declare that the research was conducted in the absence of any commercial or financial relationships that could be construed as a potential conflict of interest.
