# Supplementary material for: Gendered Development of Motivational Belief Patterns in Mathematics Across a School Year and Career Plans in Math-Related Fields
Source: Front Psychol. 2019 Jun 28;10:1472. doi: 10.3389/fpsyg.2019.01472 (PMC6610766; doi:10.3389/fpsyg.2019.01472)
Supplement: Supplementary file 1 [file Table_1.DOCX]

Supplementary Materials

*Appendix A.* Descriptive statistics

Table S1

*Descriptives of the study variables for full sample, boys and girls*

| Variable | *M* | | | *SD* | | | *Range* |
| --- | --- | --- | --- | --- | --- | --- | --- |
|  | *Total* | Girls | Boys | *Total* | Girls | Boys |  |
| Intrinsic value T1 | *3.03* | 2.85 | 3.50 | *1.12* | 1.10 | 1.09 | 1-5 |
| Intrinsic value T2 | *3.06* | 2.91 | 3.40 | *1.14* | 1.12 | 1.10 | 1-5 |
| Utility value T1 | *3.32* | 3.25 | 3.67 | *1.04* | 1.13 | 0.97 | 1-5 |
| Utility value T2 | *3.27* | 3.19 | 3.54 | *1.04* | 1.07 | 0.99 | 1-5 |
| Attainment value T1 | *3.65* | 3.67 | 3.60 | *0.99* | 1.02 | 0.92 | 1-5 |
| Attainment value T2 | *3.62* | 3.60 | 3.82 | *1.03* | 1.01 | 0.96 | 1-5 |
| Cost value T1 | *2.71* | 2.96 | 2.37 | *1.03* | 1.04 | 1.09 | 1-5 |
| Cost value T2 | *2.63* | 2.84 | 2.27 | *1.05* | 1.08 | 0.95 | 1-5 |
| Self-concept T1 | *3.16* | 3.01 | 3.47 | *0.84* | 0.83 | 0.83 | 1-5 |
| Self-concept T2 | *3.22* | 3.08 | 3.54 | *0.86* | 0.82 | 0.81 | 1-5 |
| Math career plans T1 | *43.48* | 41.42 | 45.25 | *9.60* | 9.31 | 9.89 | 1-100 |
| Math career plans T2 | *43.45* | 41.60 | 45.35 | *9.68* | 9.01 | 10.15 | 1-100 |

*Appendix B.*

Table S2a

*Model selection in the latent transition analysis*

|  | Latent classes per time point | BIC | aBIC | AIC | Entropy |
| --- | --- | --- | --- | --- | --- |
|  | 1 | 21363.35 | 21299.85 | 21270.93 | -- |
|  | 2 | 18864.39 | 18791.35 | 18758.10 | 0.857 |
|  | 3 | 17962.53 | 17841.87 | 17786.92 | 0.873 |
|  | 4 | 17647.78 | 17473.14 | 17393.61 | 0.870 |
|  | 5 | 1575.49 | 17330.51 | 17223.51 | 0.882 |
|  | 6 | 17475.01 | 17173.35 | 17035.98 | 0.884 |

*Note*. We computed latent transition models with up to eight latent classes per time point. No convergence was reached for the seven- and eight-class models. The elbow plot shows information criteria (BIC, aBIC, AIC) for models with one to six latent classes. The table shows the exact information criteria values, and entropy values. Only the information criteria were available for model selection.

In additional analyses we performed latent class analyses separately for each time point. The results suggested three to four profiles as optimal, and the profiles of the four-class model closely resembled those of the latent transition model.

Table S2b

*Model fit information for separate latent class models per time point*

|  | First half of the School Year (T1) | | | | Second half of the School Year (T2) | | | |
| --- | --- | --- | --- | --- | --- | --- | --- | --- |
| Latent classes | BIC | aBIC | AIC | p_VLMR_ | BIC | aBIC | AIC | p_VLMR_ |
| 1 | 10568.93 | 10537.17 | 10522.73 | - | 10794.42 | 10762.66 | 10748.20 | - |
| 2 | 9582.26 | 9515.57 | 9485.23 | .063 | 9731.73 | 9665.04 | 9634.68 | < .001 |
| 3 | 9228.75 | 9127.14 | 9080.91 | .001 | 9391.88 | 9290.27 | 9243.10 | .038 |
| 4 | 9173.38 | 9036.83 | 8974.71 | .787 | 9265.27 | 9128.72 | 9066.54 | .099 |

Note. p_VLMR_ = p-value of the Vuong–Lo–Mendell–Rubin likelihood ratio test.

*Appendix C.*

Table S3

*Transition probabilities between the profiles at T1 and T2 based on the estimated model*

| Transition Pattern | High | Balanced | Average | Low |
| --- | --- | --- | --- | --- |
| High | .77 | .23 | .00 | .00 |
| Balanced | .07 | .86 | .06 | .01 |
| Average | .01 | .19 | .70 | .10 |
| Low | .01 | .03 | .22 | .74 |

*Appendix D.* Longitudinal profile paths predicting mathematics career plans

Table S4

*Descriptive statistics: M (SD) of career plans by profile path*

| Transition Pattern | *M* | *SD* | *M* | *SD* |
| --- | --- | --- | --- | --- |
|  | *Career Plans T1* | | *Career Plans T2* | |
| Stable high | 48.59 | 10.59 | 49.42 | 11.53 |
| Stable balanced | 43.99 | 9.03 | 43.59 | 9.05 |
| Stable average | 41.14 | 8.31 | 40.13 | 8.60 |
| Stable low | 41.06 | 7.46 | 41.36 | 7.86 |

*Note*. Due to the small sample sizes in the profile change paths our analysis focused only on the stable profile paths.

Table S5

*Results of the Tukey HSD post-hoc test*

|  |  | Difference in means | Lower limit | Upper limit | *p* adj | Hedges' *g* |
| --- | --- | --- | --- | --- | --- | --- |
| *Model 1: First half of the School Year* | | | | | |  |
| Stable high | Stable balanced | 4.61 | 1.30 | 7.91 | 0.002 | 0.45 |
| Stable high | Stable average | 7.45 | 4.02 | 10.89 | 0.000 | 0.82 |
| Stable high | Stable low | 7.53 | 2.03 | 13.04 | 0.003 | 0.76 |
| Stable balanced | Stable average | 2.85 | 0.19 | 5.50 | 0.030 | 0.33 |
| Stable low | Stable balanced | -2.93 | -7.99 | 2.13 | 0.443 | -0.33 |
| Stable average | Stable low | -0.08 | -5.23 | 5.06 | 1.000 | -0.01 |
| *Model 2: Second half of the School Year* | | | | | |  |
| Stable high | Stable balanced | 5.83 | 2.28 | 9.37 | 0.000 | 0.60 |
| Stable high | Stable average | 9.28 | 5.55 | 13.01 | 0.000 | 0.96 |
| Stable high | Stable low | 8.06 | 2.73 | 13.39 | 0.001 | 0.77 |
| Stable balanced | Stable average | 3.46 | 0.67 | 6.25 | 0.008 | 0.39 |
| Stable balanced | Stable low | -2.24 | -6.95 | 2.48 | 0.612 | -0.25 |
| Stable average | Stable low | -1.22 | -3.64 | 6.08 | 0.916 | -0.14 |
| *Note.* Model 1: 347 observations deleted due to missingness (due to students in profile change patterns and missing data on mathematics career plans). Ns for this analysis: Stable High (n = 69), Stable Balanced (n = 175), Stable Average (n = 136), Stable Low (n = 24).  Model 2: 358 observations deleted due to missingness. Ns for this analysis: Stable High (n = 61), Stable Balanced (n = 177), Stable Average (n = 125), Stable Low (n = 30). | | | | | | |
